# 3D engineered human gingiva fabricated with electrospun collagen scaffolds provides a platform for *in vitro* analysis of gingival seal to abutment materials

**DOI:** 10.1371/journal.pone.0263083

**Published:** 2022-02-03

**Authors:** Wichurat Sakulpaptong, Isabelle A. Clairmonte, Britani N. Blackstone, Binnaz Leblebicioglu, Heather M. Powell

**Affiliations:** 1 Division of Periodontology, College of Dentistry, The Ohio State University, Columbus, OH, United States of America; 2 Faculty of Dentistry, Department of Oral Medicine and Periodontology, Mahidol University, Bangkok, Thailand; 3 Department of Materials Science and Engineering, The Ohio State University, Columbus, OH, United States of America; 4 Department of Biomedical Engineering, College of Engineering, The Ohio State University, Columbus, OH, United States of America; 5 Research Department, Shriners Children’s Ohio, Dayton, Ohio, United States of America; University of California Riverside, UNITED STATES

## Abstract

In order to advance models of human oral mucosa towards routine use, these models must faithfully mimic the native tissue structure while also being scalable and cost efficient. The goal of this study was to develop a low-cost, keratinized human gingival model with high fidelity to human attached gingiva and demonstrate its utility for studying the implant-tissue interface. Primary human gingival fibroblasts (HGF) and keratinocytes (HGK) were isolated from clinically healthy gingival biopsies. Four matrices, electrospun collagen (ES), decellularized dermis (DD), type I collagen gels (Gel) and released type I collagen gels (Gel-R)) were tested to engineer lamina propria and gingiva. HGF viability was similar in all matrices except for Gel-R, which was significantly decreased. Cell penetration was largely limited to the top layers of all matrices. Histomorphometrically, engineered human gingiva was found to have similar appearance to the native normal human gingiva except absence of rete pegs. Immunohistochemical staining for cell phenotype, differentiation and extracellular matrix composition and organization within 3D engineered gingiva made with electrospun collagen was mostly in agreement with normal gingival tissue staining. Additionally, five types of dental material posts (5-mm diameter x 3-mm height) with different surface characteristics were used [machined titanium, SLA (sandblasted-acid etched) titanium, TiN-coated (titanium nitride-coated) titanium, ceramic, and PEEK (Polyetheretherketone) to investigate peri-implant soft tissue attachment studied by histology and SEM. Engineered epithelial and stromal tissue migration to the implant-gingival tissue interface was observed in machined, SLA, ceramic, and PEEK groups, while TiN was lacking attachment. Taken together, the results suggest that electrospun collagen scaffolds provide a scalable, reproducible and cost-effective lamina propria and 3D engineered gingiva that can be used to explore biomaterial-soft tissue interface.

## Introduction

Utilization of implants to treat completely or partially edentulous patients is exceptionally common with approximately two million dental implants placed per year [1]. The failure rate of these dental restorations is high with peri-implantitis a major factor in tissue loss. Among many factors leading to peri-implantitis is the lack of strong epithelial barrier following implant placement and the subsequent inability of the newly established gingival tissue to produce a tight seal around the implant [2]. Once the mucosal seal fails, bone breakdown at the interface (peri-implantitis) rapidly progresses compared to what is mostly observed as a chronic and slowly progressing problem for periodontal tissues [3,4]. While the bacterial components of the peri-implant disease progression have been intensively studied, work on the host response to the bacterial challenge around these devices is very limited. As a result, there has been considerable effort to study soft tissue-implant interactions and to develop materials and therapeutic strategies to prevent/treat peri-mucositis and peri-implantitis.

A number of properties, including implant surface roughness and chemistry, have been shown to control the tissue-material interaction. To assess the role of roughness and chemistry on peri-implantitis, both *in vitro* and *in vivo* models have been utilized. Moderately rough alumina or zirconia surfaces (average roughness, R_a_, 0.20–0.40 μm) were shown to promote greater gingival fibroblast attachment and proliferation versus smooth surfaces [5]. Attachment and early proliferation of human gingival keratinocytes were reported to be greater on moderately rough titanium surfaces (R_a_ 0.3–0.6 μm) [6]. Though these studies provide a greater understanding of the role of surface topography on cellular attachment and proliferation, their results are based on a single cell culture model. This simple model does not provide any information on how the material interact with a clinical gingival tissue containing both epithelium and connective tissue, thus cannot be directly translated to clinical outcomes.

Many keratinized and non-keratinized 3D human oral mucosa models have been engineered utilizing a wide variety of cell sources and scaffolds [1,7–11]. Oral mucosa has been constructed from primary cells, cell lines and immortalized human primary keratinocytes and fibroblasts with varying levels of stratification and epithelial differentiation [8–11]. Tissue engineered oral mucosa has been successfully engineered from naturally derived materials (e.g., collagen, hyaluronic acid, glycosaminoglycan) or synthetic polymers (e.g., polycaprolactone, polylactide). Acellular human cadaveric dermis, which maintains the natural architecture of dermis, has been widely used as the scaffolding material for engineered oral mucosa [12,13]. However, the inability to modify its structural characteristics, natural donor dependent variability, and the risk of disease transmission [13] limit the reproducibility and translation of these engineered tissues. Collagen type I gels have also been widely used; however, they are difficult to handle and can suffer from high levels of matrix contraction which can make long term *in vitro* studies challenging [14,15]. To overcome these challenges, type I collagen can be processed into scaffolds using a number of different techniques including electrospinning and lyophilization [16,17]. The ability to tune the structural and chemical properties of these scaffolds allows them to be modified for use in different organ systems including skin and oral mucosa, specifically gingiva. Thus, an ideal platform for oral mucosa engineering would utilize a controllable, mechanically stable, biologically active scaffold that encourages cell attachment and organization akin to the native tissue.

The first aim of this study was to assess the utility of multiple scaffold platforms to engineer the lamina propria. The second aim utilized the optimized engineered lamina propria to develop human engineered gingiva with high fidelity to native gingiva. Lastly, the ability of the engineered human gingiva to assess differences in gingival adhesion to dental implant abutment materials was examined.

## Materials and methods

### Tissue collection and cell culture

Primary human gingival fibroblasts (HGF) and keratinocytes (HGK) were isolated from surgical discard gingiva from 7 and 2 patients, respectively (Ohio State University Institutional Review Board protocol #2015H0149). Additionally, portions of the samples were embedded in Optimal Cutting Temperature compound (Tissue-Tek® O.C.T. compound, Sakura Finetek USA Inc., Torrance, CA) for cryosectioning and histological and immunohistochemical analysis. A modified direct explant method [18] was used to grow the cells. Briefly, the gingival tissues obtained from crown lengthening or gingivectomy cases were disinfected with 5% chloroxylenol (Dettol, Slough, UK) for 20 seconds then washed 2 times with HEPES buffered saline (HBS). The tissues were cut to approximately 1 mm^2^ in size and 5–7 pieces transferred to a 25 cm^2^ culture flask containing fibroblast medium (DMEM (ThermoFisher Scientific, Waltham, MA) supplemented with 4% fetal bovine serum (FBS; Gemini BioProducts, West Sacramento, CA), 5 μg/mL bovine insulin (Sigma, St. Louis, MO), 0.1 mM L-ascorbic acid-2-phosphate (Sigma), 0.5μg/ml hydrocortisone (Sigma) and 10ng/ml epidermal growth factor (EGF; PeproTech, Rocky Hill, NJ). The medium was changed every day until cells reached approximately 80% confluence. The monolayers were then trypsinized for 2–3 minutes to preferentially remove fibroblasts and passaged into new flasks at a density of 2,000 cells/cm^2^. HGFs were cultured in the fibroblast medium as above. The cells remaining in the flask, predominantly HGKs, were then cultured with CnT-PR medium (CELLnTEC, Zen-Bio, Inc., Research Triangle Park, NC, USA) for another 2 days before trypsinization and seeding into new flasks at a density of 4,000 cells/cm^2^. HGKs at passage 3 and HGFs at passage 2–5 were used for all experiments.

### Engineered lamina propria

Four matrices were examined for engineering the lamina propria: electrospun collagen (ES), decellularized dermis (DD), type I collagen gels (Gel) and released type I collagen gels (Gel-R). Six pieces of lamina propria were made from each matrix. Electrospun collagen scaffolds were prepared using a 10% w/v solution of acid-soluble collagen (SEMED S; DSM Biomedical, Exton, PA, USA) in 1,1,1,3,3,3-hexafluoro-2-propanol (HFP; Sigma). The solution (5 ml) was electrospun at a potential of 30 kV onto an 8.5 cm^2^ grounding plate that was positioned perpendicular to the tip of the needle at a distance of 20 cm. The electrospun scaffolds were physically crosslinked by vacuum dehydration at 140°C for 24 hrs, then chemically cross-linked in a solution of 5 mM 1-ethyl-3-3-dimethylaminopropyl carbodiimide hydrochloride (EDC; Sigma) in 100% ethanol for 24 hrs [17]. The scaffolds were disinfected in sterile 70% ethanol for 24 hrs and rinsed thoroughly and punched into uniform 1 cm diameter circles before tissue culture [17]. Decellularized dermis (Puros® Dermis Allograft Tissue Matrix, Zimmer Biomet Dental, Palm Beach Gardens, FL) was 1 cm in diameter and 0.3–0.5 mm in thickness with the basement membrane intact. The dermis was rehydrated in fibroblast medium for 3 hours prior to seeding fibroblasts to the side opposite the basement membrane (*i*.*e*. the more porous side). Both the electrospun collagen scaffolds and the decellularized dermis were placed onto non-stick polypropylene dressing (N-terface®; Winfield Labs, Inc., Richardson, TX, USA) and then onto an inoculation sponge (Hydrosorb®; Carwild Corp, New London, CT) to promote cell infiltration and significantly enhance seeding efficiency. HGF seeded constructs are incubated on the inoculation sponge for 2 hours prior to placing the constructs into a 6-well plate for long term culture. Collagen gels (2mg/ml) were formed by serial addition of 64.5 vol.% 3.0 mg/ml PureCol® solution (Advanced Biomatrix, Carlsbad, CA), 1 vol.% 1N NaOH, 10 vol.% 10X DMEM, 24.5 vol.% fibroblast-media solution (all solutions sterile and at 4°C). Gels containing HGFs were mixed and plated into Transwell® plates (Corning Life Sciences, Tewksbury, MA) or into the bottom of standard 24-well plates resulting in gels with 5 x 10^5^ cells/cm^2^. After 6 hours of incubation, fibroblast medium was added to the wells to support cell growth. At 24 hours, gels in the 24 well plates were released from the side and bottom of the well using a sterile Pasteur pipet and transferred to a 6-well plate. Gels within the Transwell® plates remained attached to the Transwell® membrane and the wall of the insert (insert ~1cm in diameter within a 6-well plate). All matrices were inoculated with HGF at 5 x 10^5^ cells/cm^2^ and cultured for 7 days with medium exchanged daily.

Prior to daily medium exchange, digital images (Sony Cyber-shot DSC-RX100; Sony; Tokyo, Japan) of each matrix were taken with a ruler in the field of view. The area of each sample was measured using ImageJ software (NIH; Bethesda, MD, USA) and contraction was calculated for each time point/matrix type and reported as average percent original area ± standard deviation.

At the final time point (day 7), 4 mm biopsy punches were collected from each matrix and an MTT assay was performed as previously described [19]. As the HGFs were inoculated based on matrix area and the matrices contracted to differing magnitudes, the MTT data was normalized to the percent original area to account for any increase in cell density resulting from matrix contraction alone (MTT Absorbance * Area at Day 7/Area at Day 0). Data is presented as normalized absorbance ± standard deviation.

After 7 days of culture, HGF-seeded matrices were embedded into OCT™ (VWR; West Chester, PA, USA), cryosectioned at 7 μm and stained with DAPI (ThermoFisher). Sections were imaged (4 sections per block, 4 blocks per group) using an epifluorescent microscope (Eclipse 90i with DS-Qi1MC digital camera, Nikon Instruments Inc., Melville, NY). Percent cell penetration into the scaffold was measured at 5 points along each section by measuring the deepest cell along a trace versus the matrix thickness along the same trace. Autofluorescence of the scaffold was utilized to identify the top and bottom surfaces of the scaffold. Average cell penetration ± standard deviation was reported.

Additionally, the structure of the decellularized dermis, electrospun collagen and collagen gel was assessed using scanning electron microscopy (SEM). For the decellularized dermis and electrospun collagen, as-fabricated, dry constructs were adhered to the SEM stub using carbon tape (Ted Pella, Carlsbad, CA), sputter coated with gold-palladium (ACE600 Sputter coater; Leica Microsystems, Buffalo Grove, IL) and imaged using scanning electron microscopy (Apreo FEG SEM; Thermo Scientific, Hillsboro, OR) at 10kV. Collagen gels were mixed as above and cast into a 24 well plate with a glass disk at the bottom. After polymerization for 24 hours, the gels were washed with deionized water and air dried. The dried samples were adhered to a SEM stub using carbon tape and sputter coated with gold-palladium prior to imaging.

### Engineered gingiva

Engineered gingiva (EG) was fabricated following the timeline shown in S1 Fig. HGFs were seeded onto electrospun collagen scaffolds at a density of 2 x 10^6^ cells/cm^2^. All cell-seeded scaffolds were then incubated, submerged, for 4 days at 37°C and 5% CO_2_ with medium changed daily [20]. Engineered gingiva medium consisted of a DMEM base was supplemented with 1% antibiotic-antimycotic, 5 mg/ml insulin, 0.5 mg/ml hydrocortisone, 100 mM L-ascorbic acid 2-phosphate, 1 M strontium chloride, 10 mg/ml linoleic acid/albumin, 2x10^-8^ M triiodothyronine, 10 μg/ml epidermal growth factor, and 7.6x10^-7^ M progesterone [20]. HGK were then seeded at a density of 1.0 x 10^6^ cells/cm^2^ onto scaffolds seeded with the HGFs. One day following HGK seeding (Day 1), the EG was lifted at air-liquid interface by placing the constructs onto a perforated stainless-steel platform, covered with a cellulose paper (Whatman^TM^, GE Healthcare, Milwaukee, WI, USA) and a polypropylene mesh (N-terface®; Winfield Labs, Inc., Richardson, TX, USA) to prevent adhesion (S1 Fig). The engineered gingiva was cultured for a total of 14 days with the medium exchanged every other day. On culture day 3, epidermal growth factor and progesterone supplements were removed from the medium. A total of 8 EG tissues were used for this experiment.

### Histology and immunohistochemistry

For histomorphological and immunohistochemical analyses, biopsies from the engineered gingiva were collected at days 4, 7, 10, and 14. Engineered gingiva and human gingiva biopsies were embedded in OCT™ and cryosectioned at 7 μm thickness. Sections were stained with hematoxylin and eosin (H&E) and imaged with light microscopy (Leica DM2500 LED; Leica Microsystems). Brightfield images were collected using Leica Application Suite version 3.4.0 software (Leica Microsystems) with a total of 5 samples per group per time point.

Cryosections at days 10, and 14 were immunostained with anti-human laminin-332 (Lam332; Abcam, Cambridge, MA, USA), and anti-human type IV collagen (ColIV; Abcam) to identify basement membrane formation. Samples were also stained with anti-human cytokeratin-4 (KRT4; Abcam), anti-human cytokeratin-5 (KRT5; Abcam), and anti-human cytokeratin-10 (KRT10; Thermo Fisher Scientific, Waltham, MA, USA) to identify epidermal/epithelial differentiation. Additionally, sections were immunostained with anti-fibroblast antibody clone TE-7 (TE-7; Sigma) to assess fibroblast distribution and density. All samples were labeled to detect cellular DNA (DAPI; Molecular Probes, Eugene, OR, USA). Sections were examined by epifluorescence microscopy (Nikon Eclipse 90i). To examine collagen content and morphology, sections were also stained with Picrosirius red and examined using polarized light microscopy (Leica DM2500). Under polarized light, thick, mature collagen type I fibers will appear red to orange in color while thinner, immature collagen fibers will appear green in color.

### Engineered gingival attachment to abutment materials

Engineered gingiva (EG) was fabricated as described above. After culturing for 6 days, the engineered gingiva was cut into 2x2 cm^2^ pieces. At day 7, 5-mm holes were made in the middle of each tissue by sterile biopsy punches (Uni-Punch®, Premier Dental, Plymouth Meeting, PA). Posts of dental materials were inserted into each prepared hole. Five types of the dental material (5-mm diameter x 3-mm height) posts provided in kind by Zimmer Inc. that have different surface characteristics were used, i.e., machined titanium, sandblasted-acid etched (SLA) titanium, titanium nitride-coated (TiN) titanium, ceramic, and polyetheretherketone (PEEK). The experiment was conducted with 5 repeats/group. The engineered gingiva with dental material samples (1 sample/group) were harvested by fixing in 10% formalin at day 14 for histological analysis. The dental material-engineered gingiva constructs were then dehydrated in alcohol and embedded in methylmethacrylate resin (Technovit 7200 VLC; Kulzer, Wehrheim, Germany). The implants were cut longitudinally along the dental implant in 200 μm thick sections using the cutting-grinding technique (EXAKT Apparatebau, Norderstedt, Germany), and subsequently ground and polished to a final thickness of approximately 40 μm [21,22]. Sections were stained with H&E and imaged using brightfield microscopy.

For scanning electron microscopy (SEM) analysis, the engineered gingiva with dental material samples (4 samples/group) were harvested by fixing in 4% paraformaldehyde for 1 hour at day 14 then washed 3 times with PBS. The method of removing dental material posts was carried out as previously described [23]. After fixing, the samples were immersed in 0.01 M PBS containing 5% EDTA and 4% sucrose for 1 day. The dental posts were then easily removed from the tissues by using forceps. After removing the dental posts from the engineered gingiva, the materials were adhered to Al stubs using conductive carbon tape (Ted Pella) and imaged using scanning electron microscopy (Apreo FEG SEM; Thermo Scientific).

### Statistical analyses

All data was analyzed using SigmaPlot 13.0 (Systat Software Inc; San Jose, CA, USA). One-way ANOVA with Tukey post-hoc tests were performed with p < 0.05 considered significant.

## Results

### Engineered lamina propria

**Fig 1:**
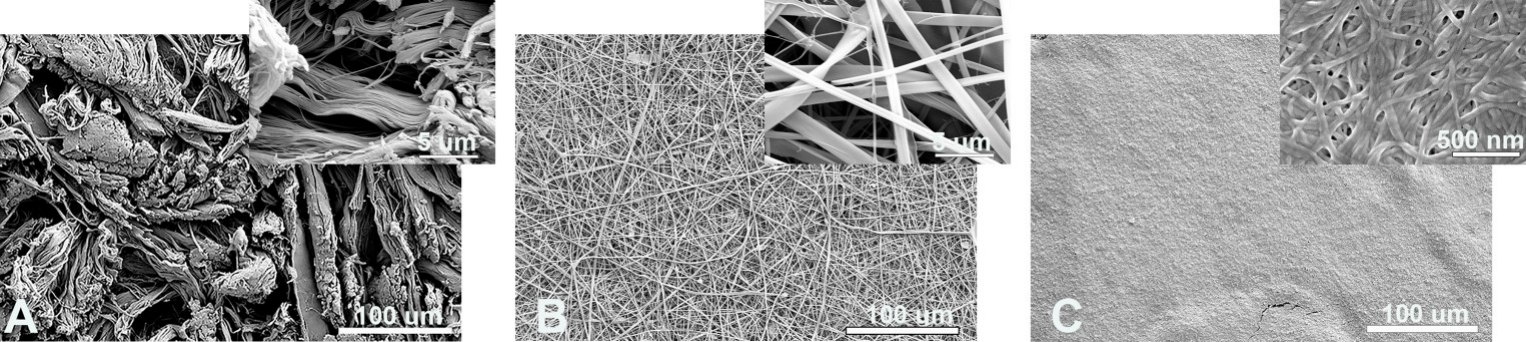
Structure of matrices for engineered lamina propria. Scanning electron micrographs of A) decellularized dermis (DD), B) electrospun collagen (ES), and C) collagen gels. Decellularized dermis was comprised of collagen fiber bundles woven into a crosshatched pattern (A), whereas electrospun collagen was nonwoven with larger individual collagen fibers. Collagen gels were dense in structure, containing smaller collagen fibers. Note the scale bar on the inset is 500 nm versus 5 μm in the insets for DD and ES matrices.

The decellularized dermis was comprised of bundles of collagen fibers woven in a crosshatch pattern (Fig 1A). Within an individual bundle of collagen there is little free space; however, between bundles of collagen there are larger more open areas. In contrast, the electrospun collagen scaffold are comprised of larger, nonwoven collagen fibers with relatively uniform free space between adjacent fibers. Collagen gels have very fine collagen fibers which could only be visualized at very high magnification (Fig 1C, inset).

**Fig 2:**
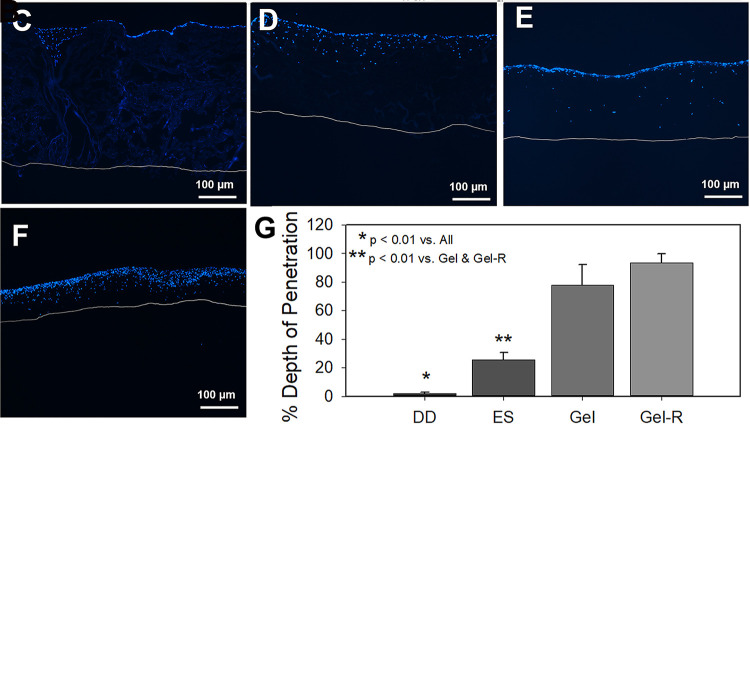
Lamina propria engineered with different scaffold systems. Multiple scaffold systems were considered for the development of human engineered gingiva including decellularized dermis (DD), electrospun collagen (ES), collagen gels cast over a membrane (Gel) and collagen gels cast into a standard culture plate and released (Gel-R). A) Contraction of engineered lamina propria as a function of culture time. B) Normalized MTT cell viability assay at day 7. DAPI stained cryosections of engineered lamina propria after 7 days of culture on C) decellularized dermis, D) electrospun collagen, E) collagen gel, and F) collagen gels that were released. White line indicates the bottom of each substrate. G) Quantification of the depth of penetration into each scaffold.

The engineered lamina propria visibly reduced in size starting from the first day after HGF inoculation onto the matrices with the most apparent contraction observed in the released gels between days 1 and 2. Little to no change in matrix size was observed in the decellularized dermis group. Quantification of matrix size confirmed visual assessment with significant reductions in area in the electrospun collagen (ES), collagen gel (Gel) and released collagen gel (Gel-R) groups at day 2 with no additional reduction in area observed between days 2 and 7 (Fig 2A). As anticipated, the Gel-R group exhibited the most contraction followed by the gel group which appeared to contract enough to separate from the wall of the Transwell® insert but were held in place by the membrane at the bottom of the insert. By day 7, the DD group had not contracted, with the ES and Gel groups contracting to 75–80% original area and the Gel-R group contracting significantly more to approximately 20% original area (Fig 2A).

A significant decrease in cellular metabolism was observed in the Gel-R group versus all other groups and the Gel group versus DD (Fig 2B). No difference in cell viability was found between ES and DD groups.

The penetration of cells into each matrix and distribution of cells within the matrix was assessed. In all matrices, HGFs were concentrated in the upper regions with HGFs present throughout the thickness of both gel groups but at reduced densities in the lower region (Fig 2C–2F). Quantification of the penetration depth showed that HGFs only penetrated into upper most regions of the DD matrix while they penetrated into the upper quarter to third of the electrospun scaffolds (Fig 2G).

### Histomorphological analysis

**Fig 3:**
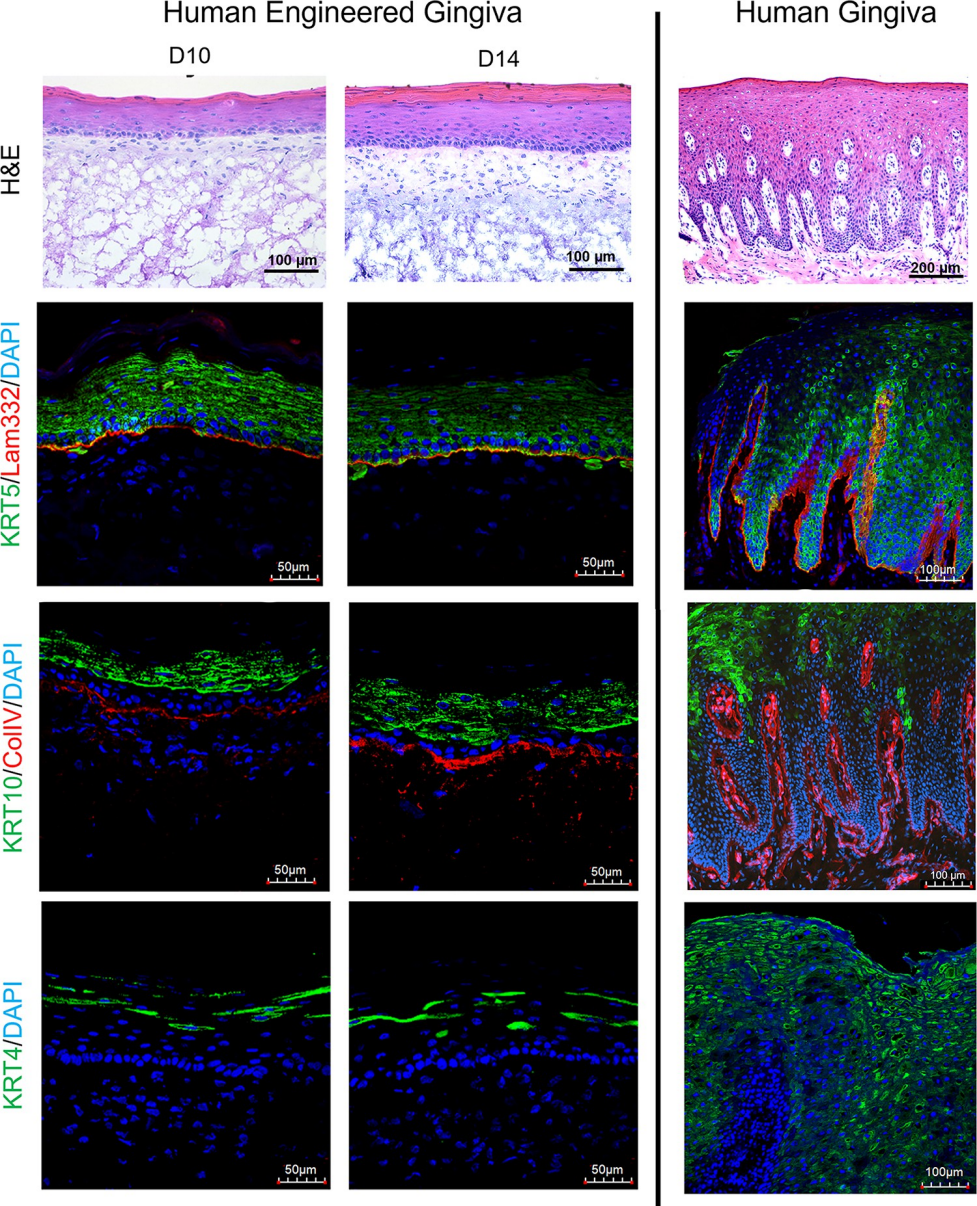
Comparison of cytokeratin and basement membrane protein expression between normal and engineered human engineered gingiva. Immunostained sections of normal human and engineered human gingiva at culture days 10 and 14. Scale bar = 50 μm for engineered gingiva and 100 μm for all native human gingiva to capture the entire epidermis.

Engineered human gingiva were found to have similar histologic appearance to the native normal human gingiva. At day 4, a clear demarcation between the epithelial and stromal tissue layers was not clear; however, by day 7 this was evident in all tissues (S2 Fig). Engineered gingiva was stratified into 2 distinct layers of epithelium and connective tissue after day 10 of culture (Fig 3). A layer of tightly packed basal keratinocytes was present along the junction between the epithelium and stromal tissue at day 10 and 14 (Fig 3). Stratum corneum was observed at the top of the epidermis by day 7 and became thicker with time (S2 Fig). In contrast to the native human gingiva, there were no rete ridges present with the overall epithelium approximately a third of the thickness of the native gingiva.

### Immunohistochemical analysis

To assess differences in epithelial structure and differentiation, immunohistochemistry was performed. Positive staining of cytokeratin-5 (KRT5), an intermediate filament protein that aids in cell-cell attachment of epithelial cells and anchors the epithelium to underlying layers of gingiva, was observed in both engineered gingiva at day 10 and 14 as well as in the native normal gingiva (Fig 3). Laminin-332 (Lam332), a major protein component of the basement membrane, showed positive staining at the junction between epithelium and connective tissue in the engineered gingiva from day 10 to day 14; and showed positive staining at the similar place in the native human gingiva (Fig 3). Collagen type IV (ColIV), a main collagen component of the basement membrane, and cytokeratin-10 (KRT10), a keratin intermediate filament protein in the post-mitotic suprabasal keratinocytes, were also present at day 10–14 in engineered gingiva and native normal gingiva (Fig 3). Positive ColIV staining is visible at the basal lamina in the engineered and native gingiva with additional staining within the lamina propria in normal gingiva where blood vessels are present. No blood vessels were present within the engineered gingiva. Positive staining of cytokeratin-4 (KRT4), a protein present during differentiation of epithelial tissues specifically in mucosal stratified epithelium, was found in the engineered gingiva at days 10 and 14 as well as in the native normal gingiva (Fig 3).

**Fig 4:**
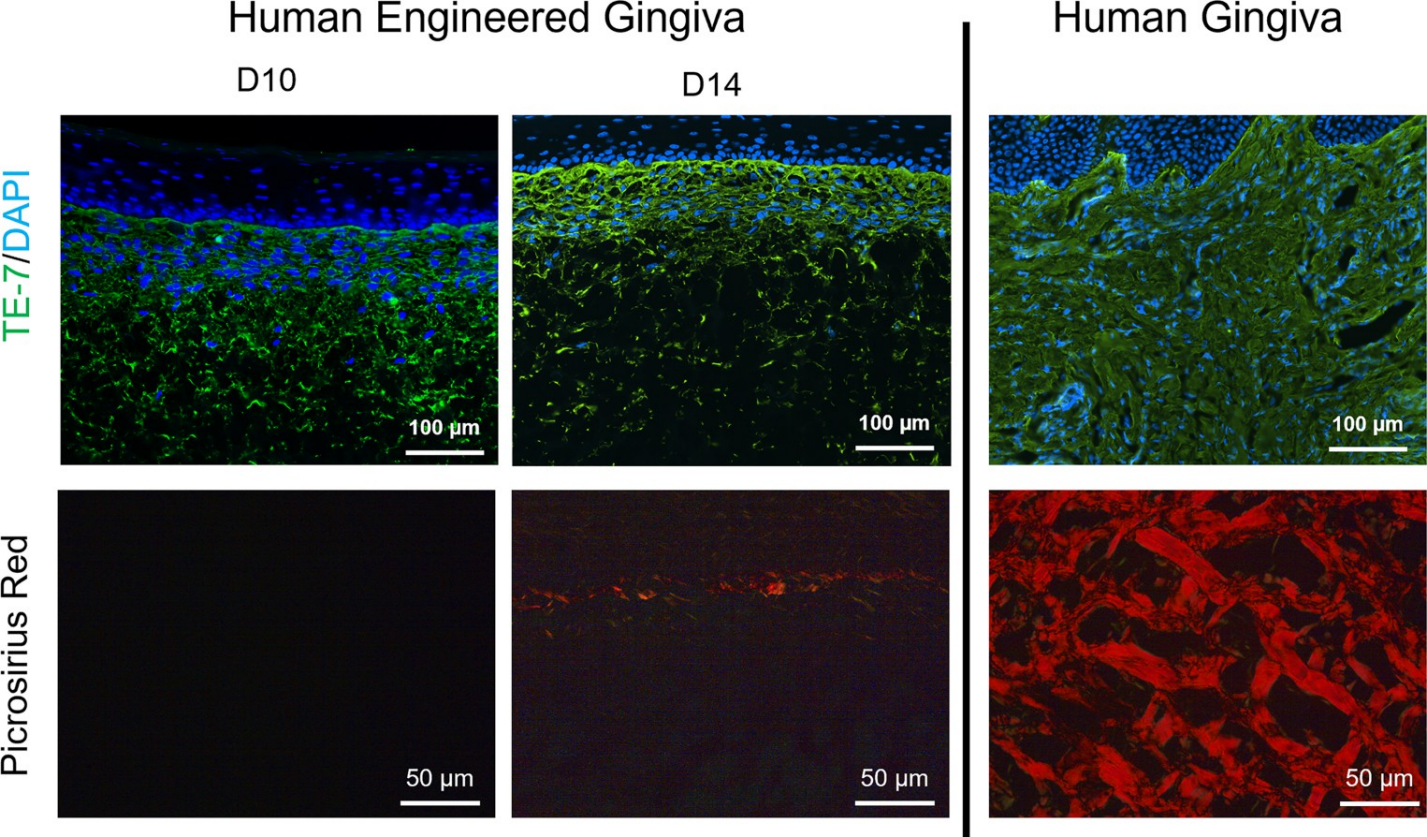
Comparison of stromal constituents in normal and engineered human gingiva. at Both normal and engineered gingiva showed dense fibroblast layers (positive staining for TE-7); however, gingival fibroblasts populated the upper half of the connective tissue scaffold in the engineered tissue whereas they were present throughout the stromal component in normal gingiva. Dense, mature collagen type I bundles (red staining) are observed in a cross-hatched pattern in normal human gingiva while new collagen has just begun to be deposited in the upper stromal region at day 14 in the engineered gingiva. Scale bar = 100 μm for TE-7 stain and 50 μm for picrosirius red staining.

Similar populations of HGFs, TE-7^+^ cells, were visible in the stromal component of both the engineered gingiva and native human gingiva (Fig 4). The distribution of the fibroblasts was more even within the native gingiva whereas these cells were concentrated near the epithelium-connective tissue junction in the engineered gingiva. Large bundles of type I collagen with a basketweave morphology were visible within the native gingiva; however, large bundles of collagen were not apparent in the engineered gingiva at day 10 and only a small layer of collagen type I fibers at the epithelium-connective tissue junction was present after 14 days in culture (Fig 4).

### Engineered gingiva attachment to abutment materials

**Fig 5:**
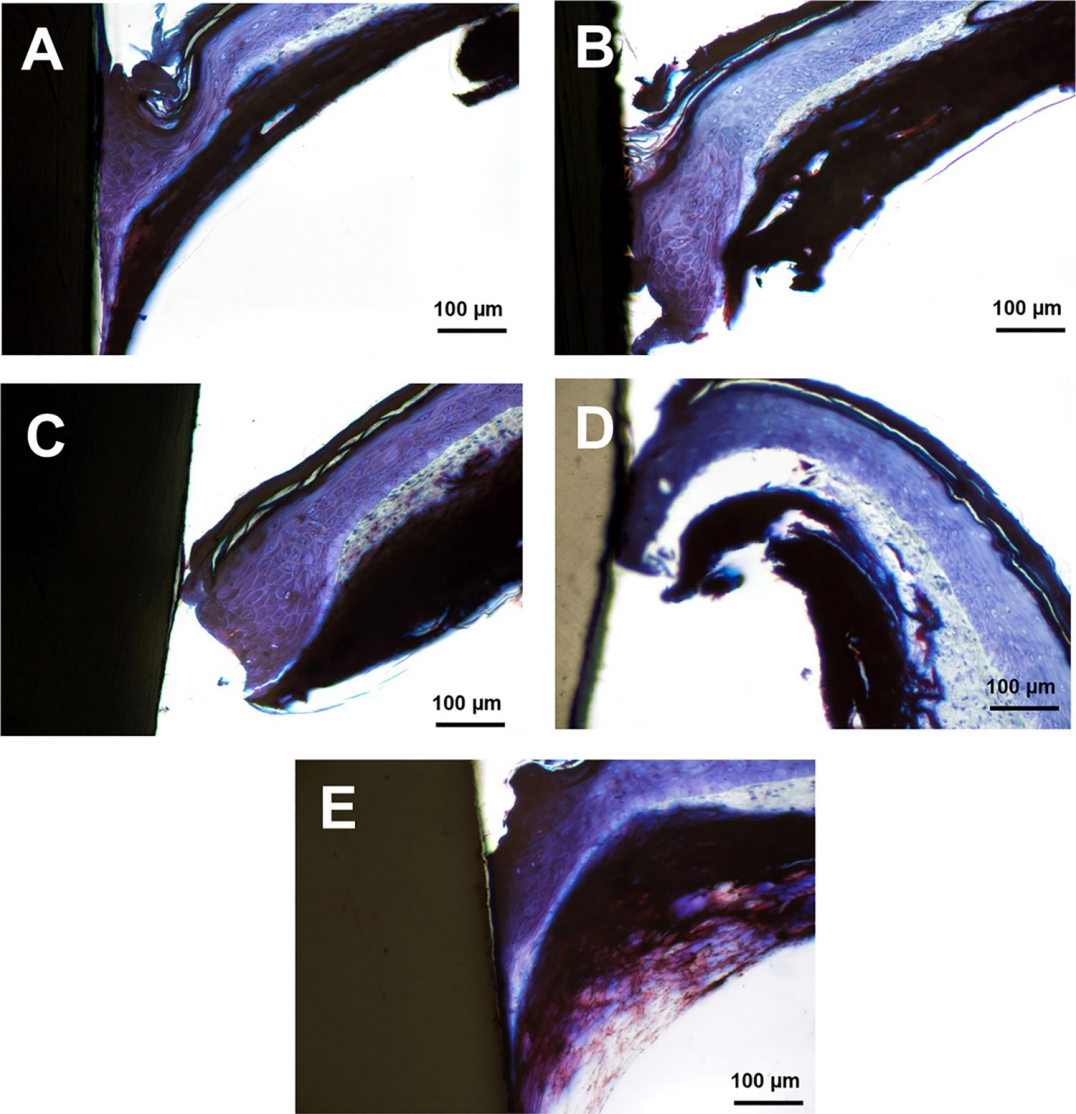
In vitro attachment of human engineered gingiva to abutment materials. H&E-stained ground sections of different abutment materials “implanted” into the engineered gingiva after 7days in culture. A) Machined commercially pure Ti, B) SLA surface modified Ti, C) TiN, D) Zirconia and E) PEEK implants exhibit a wide range of engineered gingival adhesion to the engineered gingiva. Machined Ti and PEEK have significant engineered tissue attachment to the abutment while the zirconia and TiN show weak attachment. No engineered gingiva attachment was observed with the TiN materials. Scale bar = 100 μm.

**Fig 6:**
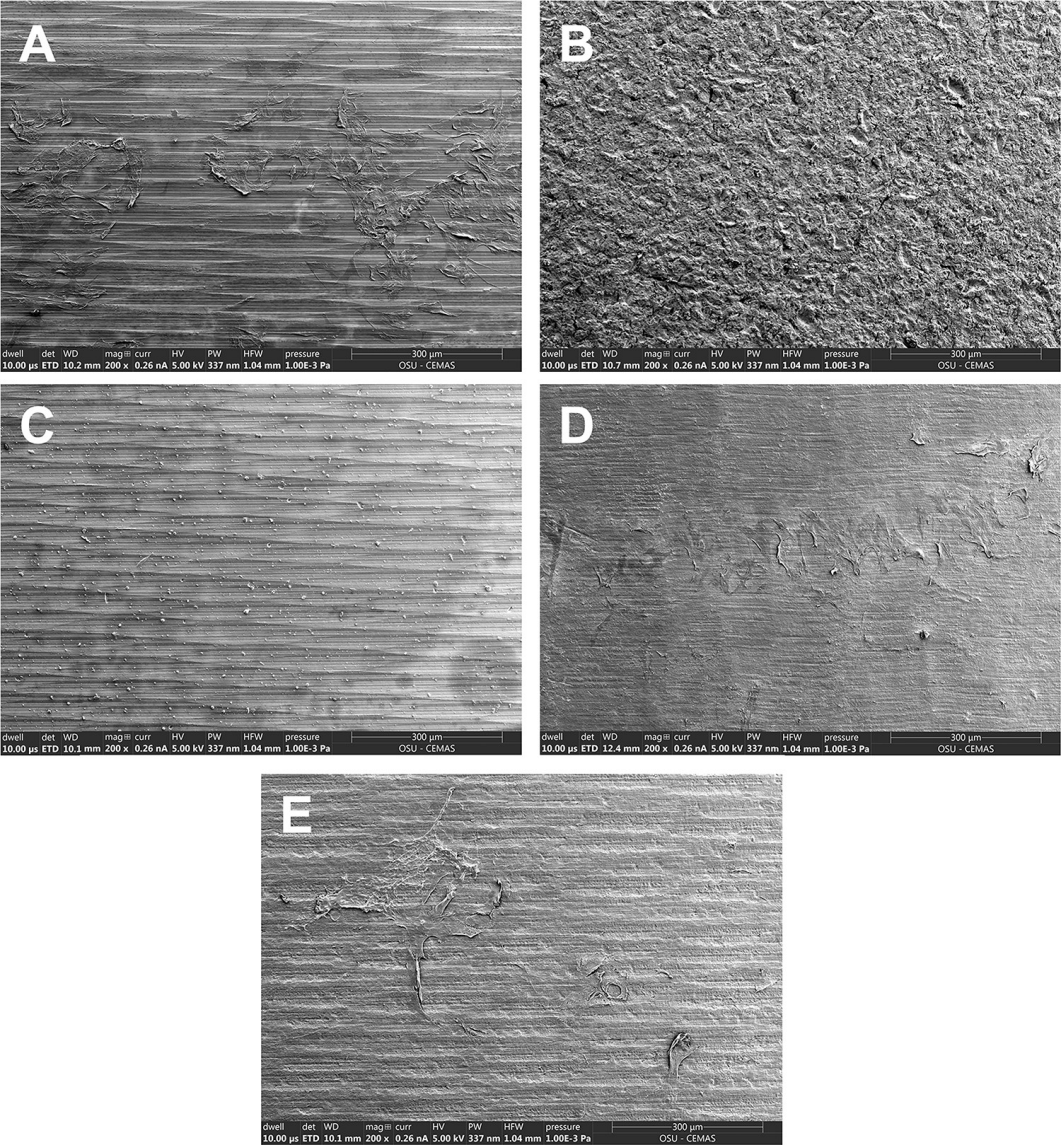
Residual tissue attachment to abutment materials after removal from human engineered gingiva. Scanning electron micrographs of abutment posts following implantation into human engineered gingiva for 7 days. A) Machined CP Ti, B) SLA surface modification, C) TiN, D) Zirconia and E) PEEK implants. Residual cellular attachment to the abutment surface following abutment retrieval was observed in the machined CP Ti, zirconia and PEEK groups. Scale bar = 300 μm.

Histological assessment of the soft tissue-implant interface at day 14 of culture showed epithelial and stromal tissue migration to the implant-gingival tissue interface in machined, SLA, Zirconia, and PEEK groups. Both the machined Ti and PEEK groups had attachment of the epithelial and stromal components while the SLA and Zirconia surfaces primarily exhibited epithelial attachment. No attachment was found in TiN (Fig 5). Examination of residual cells on the abutment materials following removal showed a thick band of cells in the machined Ti groups with a thinner band of cells present on the zirconia implant (Fig 6). Cells were visible on the retrieved PEEK implant within a wider but less dense band. No cells were clearly visible on the TiN surface (Fig 5C).

## Discussion

For greatest utility of a 3D *in vitro* tissue model, the model must be stable *in vitro*, faithfully replicate the natural tissue and be reproducible. Many collagen-based scaffolds have been utilized to form engineered tissues with benefits and challenges associated with each type. Collagen gels, electrospun collagen and decellularized matrices were all shown to support attachment as has been reported in many prior studies [8,10,11,24,25]. Cell distribution throughout the matrix was vastly different among the groups. Though the collagen gels had the smallest effective pore size of all the matrices tested, the cells were incorporated into the matrix prior to polymerization thus the cells “penetrated” the entire gel. In contrast, the decellularized dermis had tight bundles of collagen in a woven pattern leading to little infiltration in some areas and more infiltration in the inter-bundle regions. The electrospun collagen scaffolds had large individual collagen fibers with relatively homogeneous free space between the fibers. These differences in structure alter the ability of the HGFs to penetrate the scaffolds. In prior studies, scaffold pore size was found to have significant effects on cell adhesion and proliferation [26–28]. For example, fibroblasts could bind to a matrix with pore size ranged from 63–150 μm [28] and engineered skin tissue development was unsuccessful when pore size was not in a range of 20–125 μm [29]. On electrospun gelatin scaffolds, dermal fibroblasts remained mostly surficial when scaffolds had an interfiber distance of 2 μm but penetrated the entire scaffold when the interfiber distance was increased to ~12 μm [30]. Despite the difference in the cell penetration, gingival fibroblasts spread and adhered onto/into each scaffold in a similar pattern, condensed in the superficial portion and sparse in the deeper portion of the scaffold.

Tissue engineering using collagen gel has been shown to result in significant tissue contraction over time of culture, which limits this method for a long-term *in vitro* study [14,15]. Collagen gels seeded with adult human skin fibroblasts shrunk to approximately 25–40% original area after only 4 days of culture [14]. The use of crosslinked electrospun collagen matrices reduced the tissue contraction over the culture time compared to the collagen gel matrices. In the current study, scaffolds seeded with gingival fibroblasts contracted most significantly during the first two days of culture but plateaued by day 3 to approximately 73% original area, allowing for longer periods of assessment. Though the DD group did not contract at all, the ability to tune the properties of the ES scaffold combined with high cell viability and penetration into the scaffold and cost-efficiency, led this scaffold to be an optimal ECM mimic for the engineered gingiva.

Multiple studies have previously described developing an engineered gingiva tissue *in vitro* [25,31–34]. However, some deficiencies when compared to native human gingiva were observed, including undifferentiated epithelial layers [25,31–33] and the absence of a basal cell layer, basement membrane formation, and/or stratum corneum [31,34]. Engineered gingiva (EG), produced using the scalable and high throughput tissue engineering platform, was shown to develop and maintain the gingival tissue phenotype. By culture day 7, the epithelial and stromal tissue layers were found to stratify into 2 distinct layers of epithelium and lamina propria structures with basal cell differentiation and basement membrane formation similar to what is observed in healthy gingiva histology [35]. In addition, the EG expressed laminin-332, collagen type IV, cytokeratin-4, 5, and 10 similar to the expression in human normal gingiva [36–39]. Though cytokeratin-4 is most commonly observed in the suprabasal cells in all inflamed gingiva [40], Ouhayoun *et al*. demonstrated that the cytokeratin-4 could be expressed in the upper strata of healthy gingival epithelium but not expressed in healthy palatal epithelium [41].

Although the majority of 3D oral mucosa models are non-keratinized [10,11,42–45], keratinized gingiva is needed to study a healthy soft-tissue implant interface. Several studies show keratinized gingival tissue around dental implants is necessary to maintain peri-implant tissue health and prevent peri-implant disease. A five-year observation of the soft tissue around implants supporting full-arch mandibular fixed prostheses and a cross sectional study to observe the soft tissue health around implants supporting overdentures found that an inadequate keratinized gingival tissue band (< 2 mm) around the dental implants was associated with higher plaque accumulation, gingival bleeding, and soft tissue recession [46,47]. Histologically, our engineered gingival model was shown to be keratinized. In addition, the barrier function of this engineered gingiva was quantified on a macroscale using a NOVA dermal phase meter (DPM 8003; NOVA Technology Corporation, Portsmouth, NH) which measures surface electrical capacitance. These measurements revealed that by day 14 of culture our engineered gingiva reached levels of surface hydration equivalent to normal human gingiva [48].

Different soft tissue adhesion was found on the engineered gingiva-abutment material model based on the type of materials. While machined Ti and PEEK groups showed epidermal and stromal attachment, SLA and Zirconia surfaces showed primarily only epithelial attachment. Prior studies saw epithelial adhesion on polished Ti, PEEK, SLA and Zirconia materials with a slight increase in epithelial cell adhesion on polished TI and Zirconia surfaces [49]. However, most studies report fibroblast attachment to both SLA and Zirconia substrates [50–55]. In culture, gingival fibroblasts grown on SLA has significant upregulation of α-smooth muscle actin (α-SMA) compared to polished controls [56]. It is possible that SLA surface enhanced fibroblast contractility within the engineered gingiva construct causing the tissue to pull away from the post. No attachment was found in TiN. The result was also in agreement with the SEM data showing residual cell adhesion on machined Ti, zirconia, and PEEK but no presence of cells on TiN. Similar to our result, several studies also showed good fibroblast and epithelial cell adhesion on machined, SLA Ti, zirconia, and PEEK [50–55]. However, previous data on soft tissue attachment on TiN-coated abutment are still limited. Most studies tested the TiN on a single cell level and found good fibroblast attachment on the TiN material [57–59]; however, to our knowledge, a testing using 3D-engineered tissue is still not present. Our result is in contrast with the single cell experiments finding since we found no adhesion on TiN. As our model includes epithelial cells that interact with fibroblasts, the lack of attachment could be because the material was not compatible with the epithelium as it showed with the fibroblasts. Further investigation is needed to understand the soft tissue adhesion on this material.

Epithelium of the engineered gingiva was found to have similar histomorphology and immunohistochemical properties to normal human gingiva. In contrast, the lamina propria was markedly difference between the engineered tissue and native tissue which could be a result of slow remodeling of the matrix in the *in vitro* culture system. This has been observed previously where *in vitro* engineered skin epithelium was very similar to native human epithelium with the engineered dermal component resembling native human dermis only after remodeling *in vivo*. A similar result has also been observed in native oral tissues which show that gingival epithelium grows and develops faster than connective tissue or the lamina propria [60–62].

With minor levels of tissue contraction and high reproducibility, our engineered gingiva-abutment material model is a promising model that can be utilized for observing tissue attachment and integrity around different materials as well as testing factors or new drugs that may affect the soft tissue integrity.

## Conclusions

Engineered human keratinized gingival mucosa with high levels of anatomic similarity to native human gingiva, with the expression of gingiva-specific keratins, can be manufactured using a highly tunable electrospun collagen scaffold platform. This method for reproducible fabrication of 3D engineered gingiva has utility for studying implant-soft tissue interactions and shows differential adhesion based on material type and surface properties.

## Supporting information

S1 Fig Timeline for fabrication of engineered gingiva and analysis of structure and deal to abutment materials. (TIF)

S2 Fig Engineered gingiva development as a function of culture time. Biopsies were collected from engineered gingiva at days 4, 7, 10 and 14, cryosections and H&E stained. Clear stratification between the stromal and epithelial layers is evident by day 4. By day 7, a tight packed basal epithelial layer can be seen. By day 14, the epithelial layer is thick and keratinized.
(TIF)
