# Supplementary figures and images for: 3D engineered human gingiva fabricated with electrospun collagen scaffolds provides a platform for in vitro analysis of gingival seal to abutment materials
Source: PLoS One. 2022 Feb 3;17(2):e0263083. doi: 10.1371/journal.pone.0263083 (PMC8812907; doi:10.1371/journal.pone.0263083)

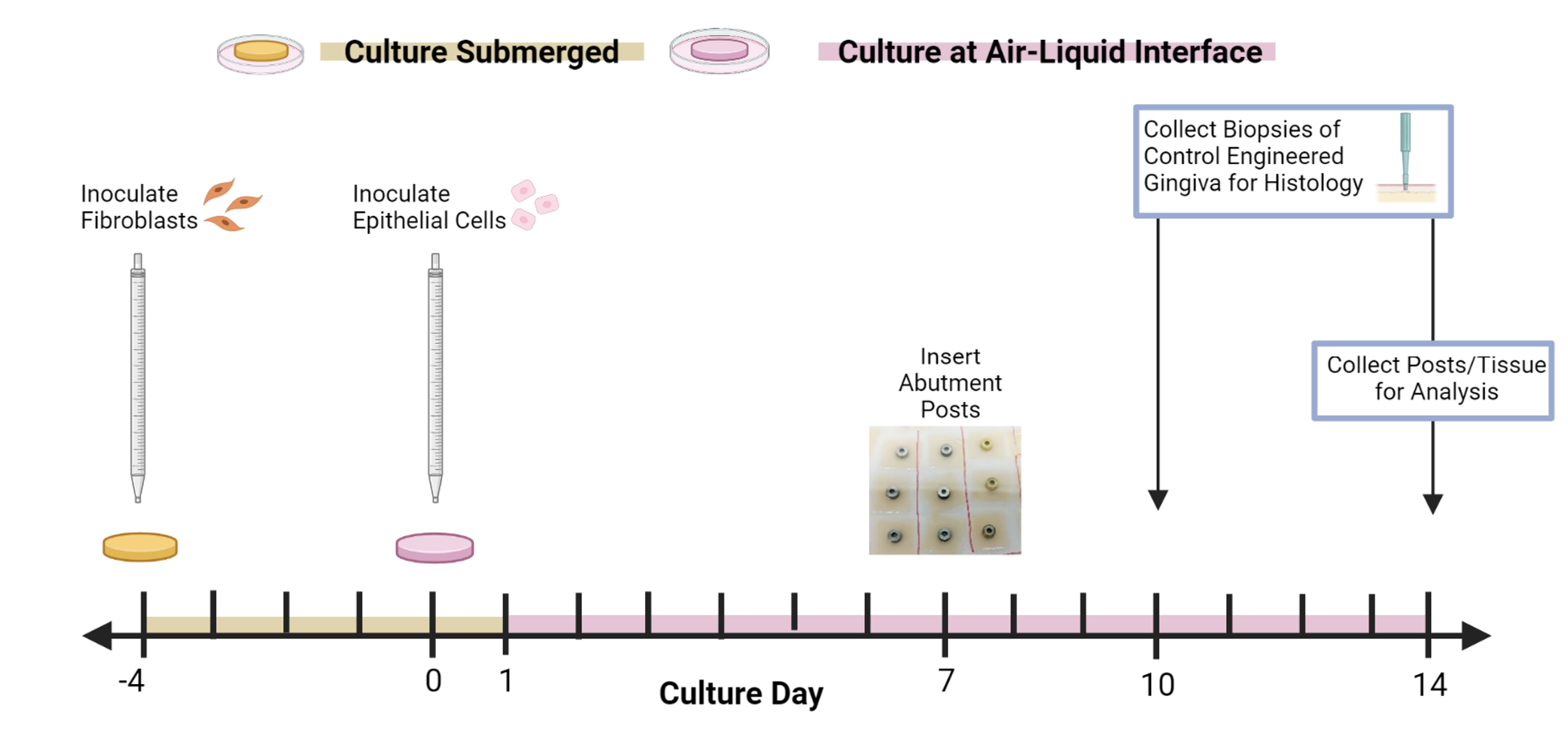

Supplement: S1 Fig — (TIF) [file pone.0263083.s001.tif]

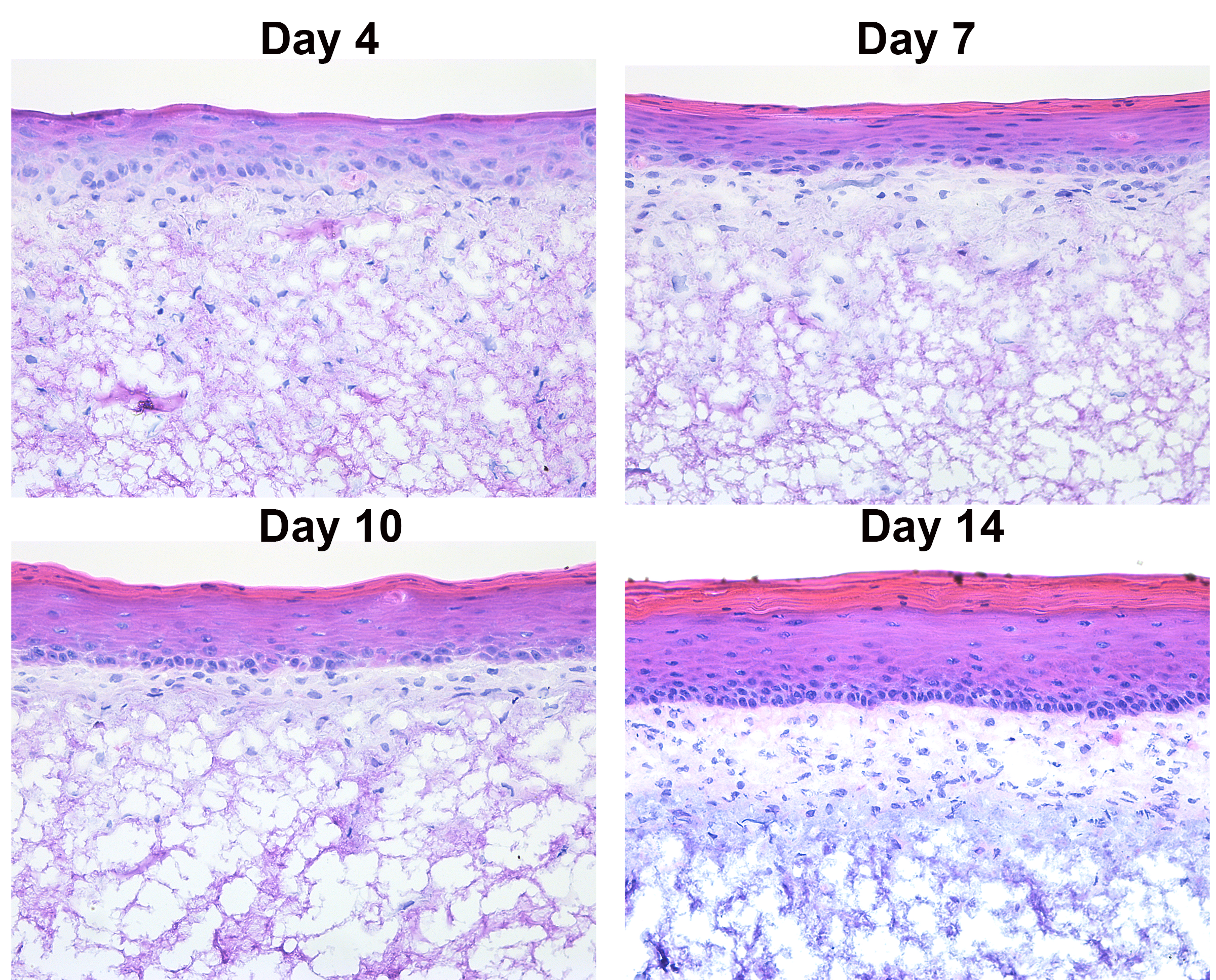

Supplement: S2 Fig — Biopsies were collected from engineered gingiva at days 4, 7, 10 and 14, cryosections and H&E stained. Clear stratification between the stromal and epithelial layers is evident by day 4. By day 7, a tight packed basal epithelial layer can be seen. By day 14, the epithelial layer is thick and keratinized. (TIF) [file pone.0263083.s002.tif]
